# Tumor Repression of VCaP Xenografts by a Pyrrole-Imidazole Polyamide

**DOI:** 10.1371/journal.pone.0143161

**Published:** 2015-11-16

**Authors:** Amanda E. Hargrove, Thomas F. Martinez, Alissa A. Hare, Alexis A. Kurmis, John W. Phillips, Sudha Sud, Kenneth J Pienta, Peter B. Dervan

**Affiliations:** 1 Division of Chemistry and Chemical Engineering, California Institute of Technology, Pasadena, California, United States of America; 2 Department of Urology, University of Michigan Medical School, Ann Arbor, Michigan, United States of America; Florida International University, UNITED STATES

## Abstract

Pyrrole-imidazole (Py-Im) polyamides are high affinity DNA-binding small molecules that can inhibit protein-DNA interactions. In VCaP cells, a human prostate cancer cell line overexpressing both AR and the *TMPRSS2-ERG* gene fusion, an androgen response element (ARE)-targeted Py-Im polyamide significantly downregulates AR driven gene expression. Polyamide exposure to VCaP cells reduced proliferation without causing DNA damage. Py-Im polyamide treatment also reduced tumor growth in a VCaP mouse xenograft model. In addition to the effects on AR regulated transcription, RNA-seq analysis revealed inhibition of topoisomerase-DNA binding as a potential mechanism that contributes to the antitumor effects of polyamides in cell culture and in xenografts. These studies support the therapeutic potential of Py-Im polyamides to target multiple aspects of transcriptional regulation in prostate cancers without genotoxic stress.

## Introduction

Pyrrole imidazole (Py-Im) polyamides are non-covalent, sequence specific DNA binders that can alter DNA architecture [1, 2]. Upon high affinity binding to the DNA minor groove, the molecules cause a 4 angstrom widening of the minor groove walls and a corresponding compression of the opposing major groove [3, 4]. Despite the relatively large molecular weight of Py-Im polyamides, these molecules are cell permeable and localize to the cell nucleus to affect endogenous gene expression [5–10]. Due to their modular sequence specificity, Py-Im polyamides can be synthesized to target DNA sequences of similar size to a protein-DNA interaction site and therefore used to antagonize gene expression driven by specific transcription factors [7, 9–13]. One such transcription factor that has been studied previously is the androgen receptor (AR) [9].

**Fig 1:**
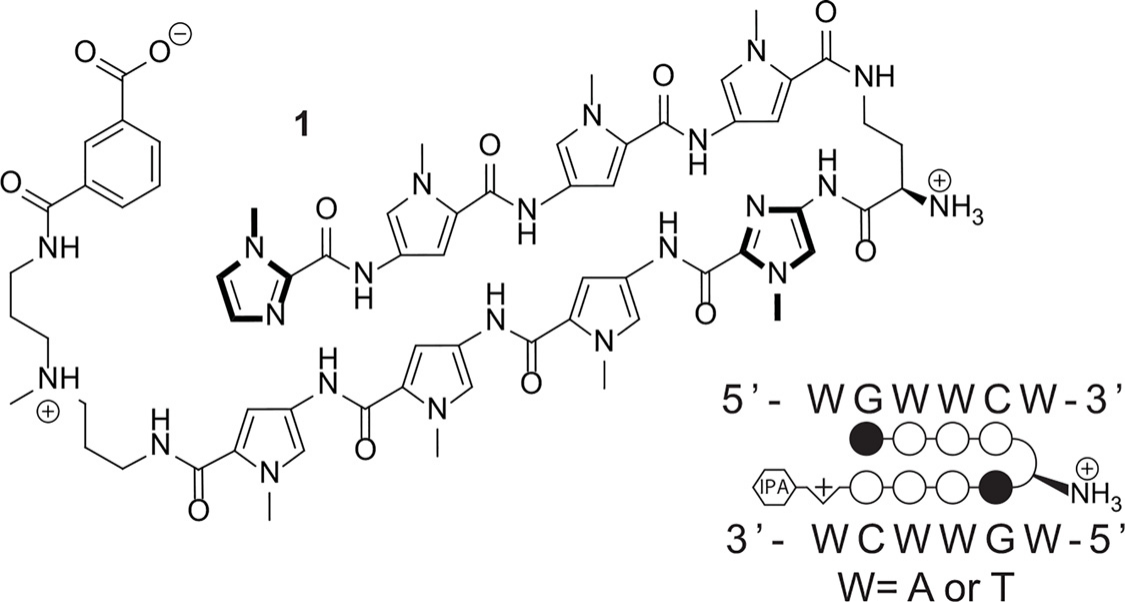
Chemical structure of a Py-Im polyamide (1) designed to target the DNA sequence 5′-WGWWCW-3′. A ball and stick notation is used to represent binding to the target DNA sequence. Pairing of an imidazole heterocycle (black circle) with a pyrrole heterocycle (white circle) allows G●C recognition, and the pairing of two pyrrole heterocycles recognizes A●T or T●A base pairs.

The AR is a dihydrotestosterone (DHT) inducible nuclear hormone receptor whose transcriptional program has been implicated in the progression of prostate cancer [14–16]. Upon ligand induction, AR will homodimerize, translocate to the nucleus and bind to conserved sequences known as the androgen response element (ARE) to regulate transcription [17]. Each monomeric unit binds to a half site of the sequence 5’-TGTTCT-3’ [18]. Polyamide **1** (Fig 1) was designed to target the sequence 5’-WGWWCW-3’ (W = A/T), found in a subset of ARE half-sites, and has been shown to prevent AR binding at select AREs and attenuate AR signaling [9].

In addition to antagonizing AR signaling, polyamide **1** is also cytotoxic towards prostate cancer cells [19]. Experiments in mice have shown that polyamide **1** is bioavailable via several routes of administration, with a serum half-life of 5.2 hours [20, 21]. In xenograft experiments, polyamide **1** has been shown to be active towards LNCaP xenografts at doses of 1 mg/kg [19]. LNCaP, however, expresses a mutated androgen receptor, and as a result, may not be representative of the majority of human disease [22]. It would therefore be useful to evaluate the efficacy of **1** against other forms of prostate cancer.

The VCaP human prostate cancer cell line expresses wild type AR and contains the *TMPRSS2-ERG* fusion [23]. Gene fusions between the *TMPRSS2* 5’-untranslated region and the *ERG* oncogene are found in approximately half of prostate cancer cases [24]. The fusion allows the AR regulated *TMPRSS2* promoter to drive the expression of *ERG*, and overexpression of *ERG* in patients has been linked with higher incidences of metastasis and poor disease prognosis [25]. In cell culture, *ERG* overexpression in immortalized prostate RPWE epithelial cells and in primary prostate epithelial cells (PrEC) has been shown to increase cellular invasiveness [26]. Due to these characteristics, the VCaP cell line presents an ideal model for the study of Py-Im polyamide activity towards this common subtype of prostate cancer. In this study, we evaluated the activity of the ARE targeted polyamide **1** in VCaP cells.

## Materials and Methods

### Synthesis and quantitation of Py-Im polyamide 1

Chemicals were obtained from Sigma Aldrich or Fisher Scientific unless otherwise noted. Synthesis was performed using previously reported procedures as indicated [7, 27]. Briefly, polyamides were synthesized by microwave-assisted solid phase synthesis on Kaiser oxime resin (Nova Biochem) [27] and then cleaved from the resin with neat 3,3’-diamino-*N*-methyldipropylamine. The triamine-conjugated polyamides were purified by reverse phase HPLC and subsequently modified at the C-terminus with isophthalic acid (IPA) or fluorescein-5-isothiocyanate (FITC isomer I, Invitrogen) [7]. The amine substituents of the γ-aminobutyric acid (GABA) turn units of the polyamides were deprotected using neat trifluoroacetic acid [28, 29]. The final polyamide was purified by reverse phase HPLC, lyophilized to dryness, and stored at -20°C. The identity and purity of the final compounds were confirmed by matrix-assisted laser desorption/ionization time-of-flight (MALDI-TOF) spectrometry and analytical HPLC. Chemical structures are represented in Fig 1 and S1 Fig Mass spectrometry characterization data are represented in S2 Fig.

Py-Im polyamides were dissolved in sterile dimethylsulfoxide (DMSO, ATCC) and quantitated by UV spectroscopy in either 4:1 0.1% TFA (aqueous):acetonitrile (ε(310nm) = 69,500 M^-1^cm^-1^) or 9:1 water:DMSO (ε(310nm) = 107,100 M^-1^cm^-1^) as dictated by solubility. Polyamides were added to cell culture solutions at 1000x concentration to give 0.1% DMSO solutions.

### Cell culture

The VCaP cell line was obtained from the laboratories of Dr. Kenneth J. Pienta and Dr. Arul M. Chinnaiyan at the University of Michigan Department of Pathology, where the cell line was derived [30]. VCaP cells were received at passage 19 and cultured in Dulbecco’s modified eagle medium (DMEM, Gibco 10313–039) with 4 mM glutamine (Invitrogen) and fetal bovine serum (FBS, Omega Scientific) on Corning CellBind flasks. All experiments were performed below passage 30.

### Cellular uptake studies

For visualization of uptake using FITC-analog polyamides, VCaP cells were plated in 35-mm optical dishes (MatTek) at 7.5×10^4^ cells per dish and allowed to adhere for 48 h. Media was then changed and cells were treated with 0.1% DMSO with polyamide for 24 or 48 h. Cells were imaged at the Caltech Beckman Imaging Center using a Zeiss LSM 5 Exciter inverted laser scanning microscope equipped with a 63x oil immersion lens as previously described [5].

### WST-1 proliferation assay

VCaP cells were plated at 2x10^4^ per well in 96-well plates coated with poly-L-lysine (BD BioCoat). After 24 h, an additional volume of medium containing vehicle or polyamide was added to each well. All medium was removed following polyamide incubation at the indicated time points and replaced with one volume of WST-1 reagent (Roche) in medium according to manufacturer protocol. After 4 h of incubation at 37°C, the absorbance was measured on a FlexStation3 plate reader (Molecule Devices). The value of A(450 nm)-A(630 nm) of treated cells was referenced to vehicle treated cells. Non-linear regression analysis (Prism software, Graphpad) was performed to determine IC_50_ values.

### Gene expression analysis by quantitative RT-PCR (qPCR)

For DHT induction experiments, VCaP cells were plated in 6-well plates coated with poly-L-lysine (BD BioCoat) in charcoal-treated FBS containing media at a density of 31k/cm^2^ (3x10^5^ cells per well). The cells were allowed to adhere for 24 h and then dosed with 0.1% DMSO with or without polyamide **1** for 72 h followed by the addition of 0.01% ethanol in PBS with or without DHT (1 nM final concentration). Cells were harvested after additional 24 h incubation. Cells treated with etoposide and camptothecin (Sigma) were co-treated with DHT (1 nM) and harvested after a 16 h incubation. For native expression experiments, VCaP cells were plated as above but using standard FBS media and harvested after 72 h of treatment. For all experiments, the mRNA was extracted using the QIAGEN® RNeasy mini kit following the standard purification protocol. Samples were submitted to DNAse treatment using the TURBO DNA-*free*™ Kit (Ambion), and the mRNA was reverse-transcribed by using the Transcriptor First Strand cDNA Synthesis Kit (Roche). Quantitative PCR was performed by using the FastStart Universal SYBR Green Master (Rox) (Roche) on an ABI 7300 Real Time PCR System. Gene expression was normalized against *GUSB*. Primers used are referenced in S3 Fig.

### Immunoblot of ERG protein levels

For assessment of ERG and beta-actin protein levels, 3x10^6^ VCaP cells were plated in 10 cm diameter dishes with charcoal-treated FBS containing media for 24 h before treatment with 0.1% DMSO vehicle with or without polyamide **1** for an additional 72 h. Ethanol (0.01%) in PBS with or without DHT (1 nM final concentration) was then added. After 24 h incubation, cells were lysed in TBS-Tx buffer (50 mM Tris-HCl pH 7.4, 150 mM NaCl, 1 mM EDTA, 1% Triton X100) containing fresh 1 mM phenylmethanesulfonylfluoride (PMSF) and protease inhibitors (Roche). The samples were quantified by Bradford assay, denatured by boiling in Laemmli buffer, and total protein was separated by SDS-PAGE. After transfer to the polyvinyl difluoride (PVDF) membrane (Bio-Rad) and blocking with Odyssey Blocking Buffer (LI-COR), primary antibodies were incubated overnight at 4°C. Rabbit monoclonal anti-ERG antibody (Epitomics 2805–1) and rabbit polyclonal anti-actin antibody (Sigma A2066) were used. Goat anti-rabbit near-IR conjugated secondary antibody (LI-COR) was added and the bands were visualized on an Odyssey infrared imager (LI-COR). The experiment was conducted in duplicate and the data are representative of both trials.

### Single cell electrophoresis (COMET) assay

VCaP cells (3x10^6^ cells) were plated in 10 cm cell culture dishes and allowed to adhere for 24 h before addition of DMSO vehicle or polyamide stock in DMSO. After 72 h incubation, cells were washed with warm PBS (37°C), gently scraped, and counted. Samples were centrifuged, resuspended at 1x10^5^ cells/mL, and treated according to manufacturer protocol (Trevigen) for neutral electrophoresis. Slides were stained with SybrGreen (Trevigen) and imaged at the Caltech Beckman Imaging Center using a Zeiss LSM 5 Pascal inverted laser scanning microscope equipped with a 5x air objective lens. Overlayed fluorescence and bright field images were obtained using standard filter sets for fluorescein. Images were analyzed using Comet IV software (Perceptive Instruments Ltd) with 200–600 comets measured per sample. A random sampling of 200 comets per condition was used for two-way analysis of variance (ANOVA) analysis (Prism software, GraphPad) of three biological replicates.

### Xenograft assays

Male severe combined immunodeficiency (SCID) mice (4–6 weeks old) were obtained from a breeding colony maintained by the University of Michigan. Tumors were induced by subcutaneous injection of 1x10^6^ VCaP cells (10 mice per dose group) in 200 μL of Matrigel (BD Biosciences, Inc., San Jose, CA) above the right flank. Tumor growth was monitored by caliper measurement until the tumor size reached 100 mm^3^ using the formula 0.56 *x L* x *W* ^2^. Groups were randomized and all mice were treated subcutaneously with control (DMSO) or with polyamide **1** as reported (3 times per week, 10 total injections). Tumor growth was followed weekly by caliper measurements. Animal husbandry and daily care and medical supervision was provided by the staff of the Unit for Laboratory Animal Medicine (ULAM) under the guidance of supervisors who are certified as Animal Technologists by the American Association for Laboratory Animal Science (AALAS) at the University of Michigan. Animals were monitored twice daily by both the research team and the veterinary staff. Health was monitored by weight (twice weekly), food and water intake, and general assessment of animal activity, panting, and fur condition. The experiments were performed in accordance with the guidelines on the care and use of animals set by the University Committee for the Use and Care of Animals (UCUCA) of the University of Michigan, and all procedures in this study were specifically approved by the UCUCA (Protocol Number 3848). In all cases, appropriate measures were taken to minimize discomfort to animals. All injections or surgical procedures were performed using sterile technique with efforts made to minimize trauma to the animals. When necessary, animals were anesthetized with a mixture of 1.75% isofluorane/air. Following injections animals were closely monitored and any that appeared moribund were immediately euthanized by administration of anesthesia, followed by inhalation of carbon dioxide until breathing ceased. Death was then ensured through cervical dislocation.

### RNA-seq analysis

VCaP cells (1x10^6^ cells) were plated in 20 cm cell culture dishes and allowed to adhere for 72 h in DMEM containing 10% FBS and 4 mM glutamine. Polyamide **1** or 0.1% DMSO vehicle were then added in fresh media and allowed to incubate for 96 h. Total RNA was collected by trizol extraction. Library building and sequencing were performed at the Caltech Millard and Muriel Jacobs Genetics and Genomics Laboratory. Sequenced reads were mapped against the human genome (hg19) with Tophat2 using Ensembl GRCh37 gene annotations [31]. Exon alignment was performed with htseq-count and differential expression was determined with DESeq2 [32, 33]. Genes with padj < 0.05 and |log_2_(fold change)| ≥ 1 were submitted for connectivity map analysis online at http://lincscloud.org.

### Topoisomerase inhibition assay

Topoisomerase inhibition kits were purchased from Topogen (Port Orange, FL). For Top2 relaxation assays, 540 ng Top2α-p170 fragment (16 units) was added to 250 ng supercoiled pHOT1 DNA in assay buffer (0.05 M Tris-HCl (pH 8), 0.15 M NaCl, 10 mM MgCl2, 0.5 mM dithiothreitol) plus 2 mM ATP with or without test compounds in a total volume of 20 μL. The DMSO concentration was standardized to 1% for all samples except the no-DMSO solvent controls. Reactions were incubated at 37°C for 30 min and then quenched with 2 μL 10% sodium dodecyl sulfate solution. Samples were then extracted with chloroform: isoamyl alcohol 24:1, mixed with 2 μL 10x glycerol loading buffer and loaded onto 1% agarose gels in tris-acetic acid-EDTA (TAE) buffer with or without 0.5 μg/mL ethidium bromide (EtBr). Gels run without EtBr were post-stained with SYBR-Gold (Invitrogen).

For Top1 assays, 0.5 μL Top1 (5 units) was added to 250 ng supercoiled pHOT1 DNA in assay buffer (10 mM Tris-HCl (pH 7.5), 1 mM EDTA) plus 2 μL reaction buffer (10 mM Tris-HCl (pH 7.9), 1 mM EDTA, 0.15 M NaCl, 0.1% BSA, 0.1 mM spermidine, 5% glycerol) with or without test compounds in a total volume of 20 μL. The DMSO concentration was again standardized to 1% for all samples except the no-DMSO solvent controls. Reactions were incubated at 37°C for 30 min and then quenched with 4 μL stop buffer (0.125% bromphenol blue, 25% glycerol, 5% Sarkosyl). Samples were then loaded onto 1% agarose gels in tris-acetic acid-EDTA (TAE) buffer with or without 0.5 μg/mL ethidium bromide (EtBr). Gels run without EtBr were post-stained with SYBR-Gold.

## Results

### Nuclear uptake and cytotoxicity of Py-Im polyamide

**Fig 2:**
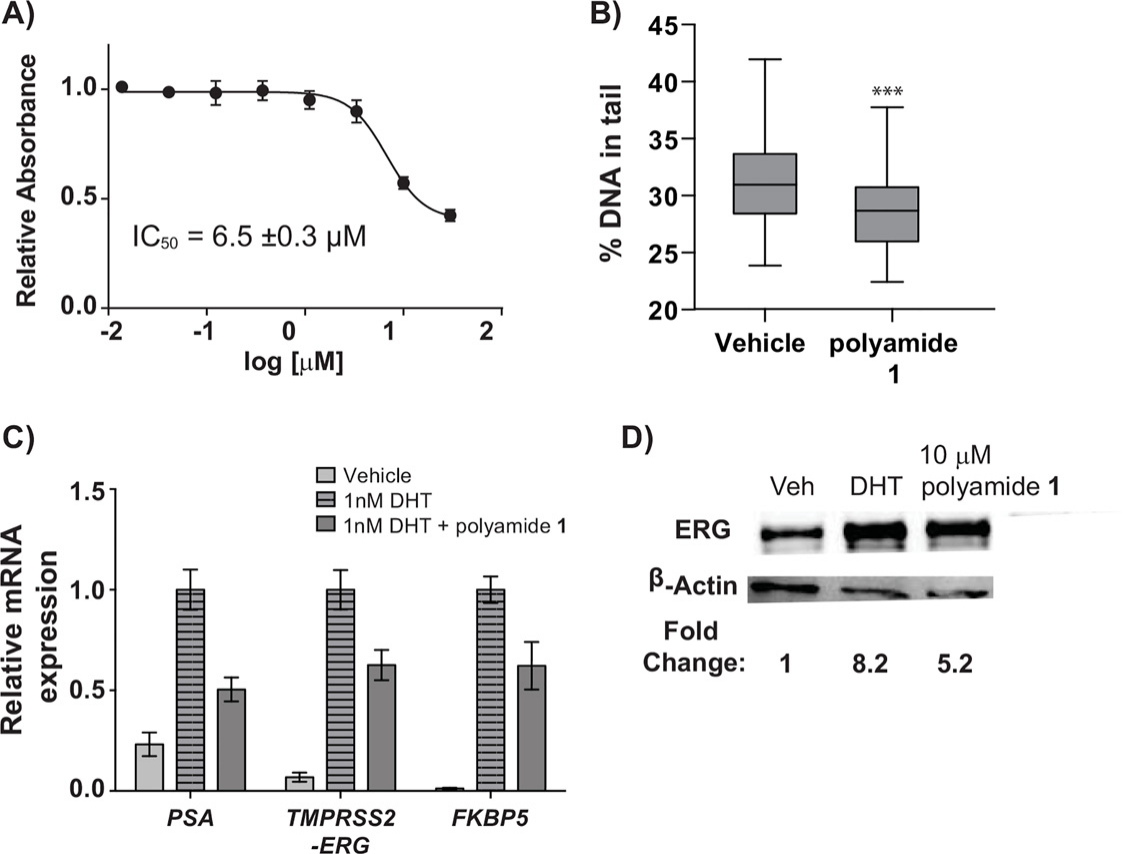
Cell culture characterization of polyamide 1 in VCaP cells. (A) Cytotoxicity of polyamide **1** in VCaP cells after 96 h incubation. (B) Quantification of 200 neutral comets from VCaP cells treated with 0.1% DMSO (vehicle) or 10 μM polyamide **1** for 72 h. Statistical significance was determined using two-way ANOVA analysis (Prism) where *** = p < 0.001 relative to vehicle. Boxes are bounded by the upper and lower quartile, while whiskers represent the 1st and 99th percentile. (C) Effect of 10 μM polyamide **1** on select androgen receptor regulated genes in VCaP cells under 1 nM DHT induction. (D) Change in ERG protein caused by 0.1% DMSO vehicle, 1 nM DHT alone, and cotreatment of 10 μM polyamide **1** with 1 nM DHT.

To test the nuclear uptake potential of polyamide **1**, a FITC-labeled derivative was prepared (**1-FITC**) and incubated with VCaP cells prior to imaging by confocal microscopy (S1 Fig). Polyamide **1-FITC** signal was observed in the nucleus and also showed significant membrane binding. The overall level of uptake in VCaP cells was found to be qualitatively less than that in LNCaP cells [21]. Next, polyamide **1** was evaluated for antiproliferation effects in VCaP cells using the WST-1 assay under conditions similar to the gene expression experiment. After a 96 h incubation with polyamide, an IC_50_ value of 6.5 ± 0.3 μM was determined for polyamide **1** (Fig 2A). At 72 h, the IC_50_ value for polyamide **1** in VCaP cells was found to be over 30 μM (data not shown). For comparison, polyamide **1** has been found to have an IC_50_ of 7 ± 3 μM after 72 h incubation in LNCaP cells [19].

### Reduction of DNA damage in VCaP cells upon treatment with Py-Im polyamide

The effect of polyamide **1** on the high level of extant DNA damage in VCaP cells was also investigated. After incubation with polyamide, VCaP cells were submitted to the neutral Comet assay, which allows visualization of double-strand breaks through single cell electrophoresis (Fig 2B). The percentage of DNA in the “tail” of the comets was then compared using two-way ANOVA statistical analysis (S4 Fig). A significant reduction in DNA damage (p < 0.001) was observed with polyamide **1** over the vehicle control.

### ARE-targeted Py-Im polyamide downregulates AR-driven *TMPRSS2-ERG* expression

Next the effect of polyamide **1** on AR signaling in ERG-positive cells was examined. Dosage concentrations were chosen based on previous reports of polyamide gene expression effects in LNCaP [28, 34]. In VCaP cells, polyamide **1** was found to reduce the DHT-induced expression of the *TMPRSS2-ERG* fusion as well as other AR target genes, including *PSA* and *FKBP5* (Fig 2C). Corresponding decreased expression of ERG protein was confirmed by Western blot (Fig 2D). In the non-induced state, polyamide **1** was also found to reduce expression of several ERG influenced genes, including *PLAT* and *MYC* (S5 Fig).

### Diminished growth in VCaP xenografts upon polyamide treatment

**Fig 3:**
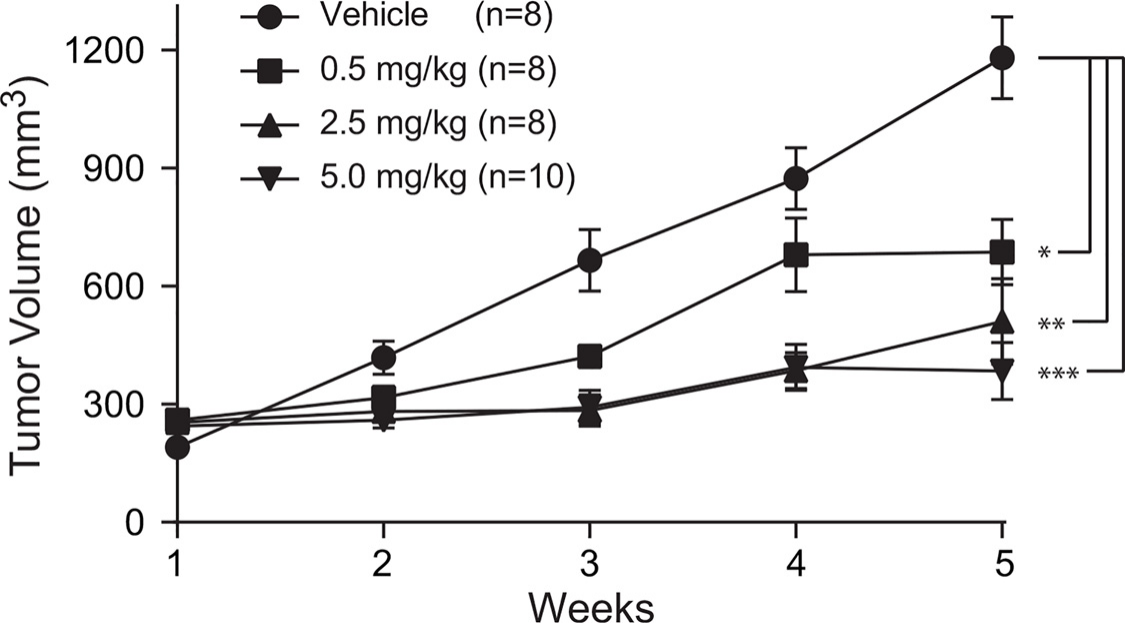
VCaP xenografts treated with polyamide 1 three times per week showed a dose dependent reduction in tumor growth. Tumor volume was determined by caliper measurements. Errors are SEM. * = p < 0.01, ** = p < 0.0005, *** = p < 0.0001.

We next moved from cell culture studies to investigations of polyamide **1** in a VCaP mouse xenograft tumor model. Xenograft experiments were conducted in male SCID mice bearing subcutaneous VCaP cell xenografts. Treatments were started after tumor sizes in each group of mice reached ~100 mm^3^ and were administered three times per week through subcutaneous injection in DMSO vehicle for three weeks for a total of 10 injections. Dose-dependent retardation of tumor growth was observed in mice treated with polyamide **1** (Fig 3). After 5 weeks of monitoring, tumors treated with vehicle grew to approximately 6-fold the initial volume of that group while tumors treated with polyamide **1** at 5.0 mg/kg grew to approximately 1.6-fold the initial volume of that cohort.

### Genome wide expression analysis

**Fig 4:**
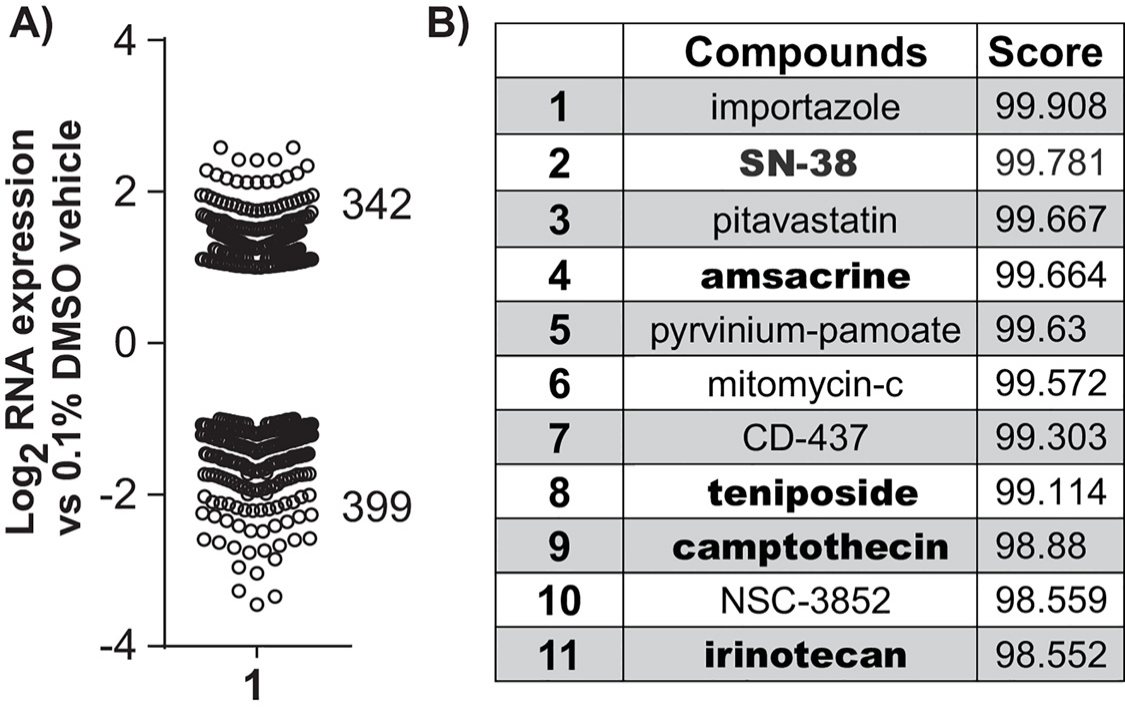
Genome wide expression analysis of VCaP cells treated with 10 μM polyamide 1 for 96 h. (A) Scatter plot of gene expression changes in VCaP cells after polyamide treatment. (B) Connectivity map analysis of perturbagens that correlate with gene expression changes induced by polyamide **1**. Bolded compounds are topoisomerase inhibitors.

RNA-seq analysis was performed after 96 hours of polyamide treatment in order to assess gene expression changes after prolonged exposure and to identify potential mechanisms of polyamide induced toxicity. Differential expression analysis using DESeq2 showed that of the genes with padj < 0.05 and |log_2_(fold change)| ≥ 1, 342 were upregulated and 399 were downregulated upon polyamide treatment (Fig 4A). Connectivity map analysis of these genes returned several compounds known to be topoisomerase inhibitors (Fig 4B), suggesting that the polyamide may also be interfering with topoisomerase activity. Analysis of a previously published genome wide data set from LNCaP cells treated with polyamide **1** shows similar results (S6 Fig)[9].

### Inhibition of topoisomerases 1 and 2

**Fig 5:**
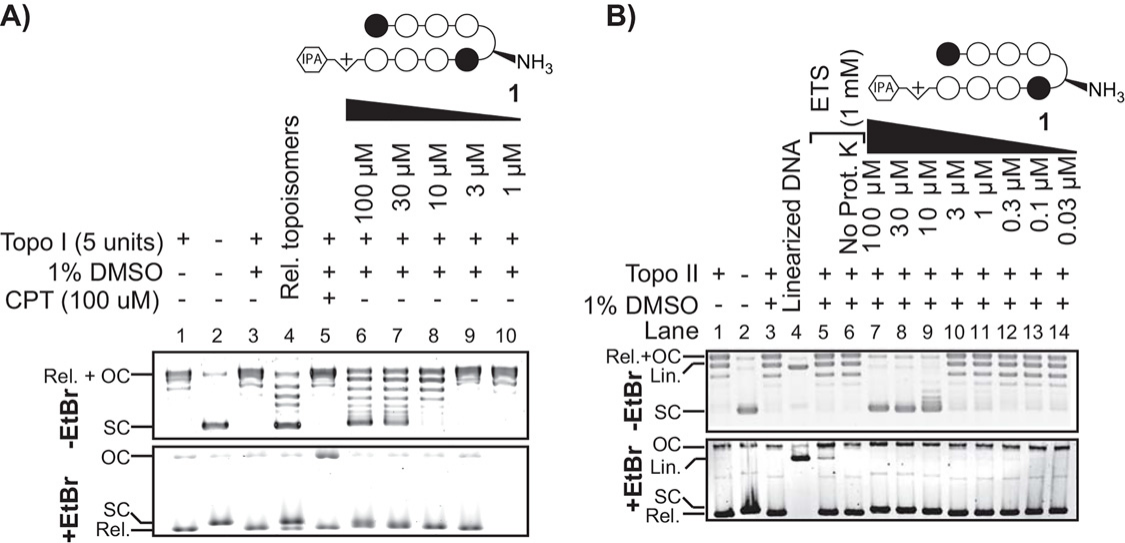
Inhibition of topoisomerase I and II by polyamide 1 in vitro. (A) Supercoiled DNA relaxation assay for topoisomerase I treated with increasing concentrations of polyamide **1**. Camptothecin (CPT) is used as a positive control. (B) Supercoiled DNA relaxation assay for topoisomerase IIα-p170 fragment. Etoposide (ETS) is used as a control. Rel, relaxed; OC, open circular; SC, supercoiled; Lin, linear; Rel. topoisomers, relaxed topoisomers; Prot. K, proteinase K.

Topoisomerase inhibitors have been shown to attenuate AR signaling in multiple cell lines [35, 36]. Similar results are also seen in VCaP cells, where treatment with etoposide and camptothecin is able to reduce DHT induced expression of select AR regulated genes (S7 Fig). Based on the Connectivity map results, we examined the inhibitory effects of polyamide **1** against topoisomerase 1 and 2 *in vitro*. Topoisomerase 1 (Topo1) functions by relieving DNA supercoils generated by transcription and replication and is a therapeutic target in cancer [37]. To determine if polyamide **1** inhibits Topo1 mediated DNA cleavage, we titrated polyamide **1** with supercoiled pHOT1 plasmid and measured conversion to open circular plasmid or relaxation upon addition of purified Topo1. A reduction in DNA relaxation indicates polyamide **1** was able to attenuate Topo1 mediated cleavage of DNA (Fig 5A). To differentiate between open circular and relaxed DNA, samples were also run on an EtBr gel. Unlike camptothecin (CMT), which traps the Topo1 cleavage complex and generates nicked open circular DNA, treatment with polyamide **1** did not prevent DNA re-ligation. Topoisomerase II cleaves double stranded DNA in an ATP dependent manner and is essential for strand separation of tangled daughter chromosomes during replication. Like Topo1, Topo2 is targeted in cancer therapy [38]. Similar to results seen for Topo1, polyamide **1** was able to inhibit Topo2 cleavage of supercoiled pHOT1 plasmid in a concentration dependent manner (Fig 5B). Furthermore, samples were run with EtBr to allow unambiguous identification of linearized DNA, which allowed the identification of Topo2 cleavage complex (Topo2cc) formation (Fig 5B, lanes 5 and 6). The lack of Topo2cc formation in polyamide **1** treated samples as compared to linearized DNA and etoposide-treated samples is consistent with disruption of Topo2 binding.

## Discussion

In this study, we evaluated the activity of an ARE targeted polyamide in VCaP human prostate cancer cells. Polyamide **1** has been previously shown to exhibit antitumor activity in cell culture and in xenografts of the androgen sensitive LNCaP cell line [19], but there are several important genotypic differences between these two cell lines. First, VCaP cells possess an amplified AR region, leading to higher levels of AR protein than LNCaP cells [30, 39, 40]. Additionally, VCaP cells belong to a subtype of prostate cancer that possesses the *TMPRSS2*:*ERG fusion*, resulting in the AR driven expression of *ERG* [23]. *ERG*, an oncogenic transcription factor, has been reported to increase double stranded DNA break formation in PrEC cells, while knockdown of *ERG* by siRNA in VCaP cells have been shown to decrease DNA breaks [41]. Studies have also shown that *ERG* overexpression increases cancer invasiveness and has been correlated to increased metastasis in the clinic [25, 26].

In VCaP cell culture experiments, polyamide **1** exhibited antiproliferative activity and attenuated the DHT induced expression of select AR driven genes including *TMPRSS2*:*ERG*. Furthermore, in this cell line with high genomic instability due to *ERG* overexpression, treatment with polyamide **1** repressed the high level of DNA fragmentation found in the basal state, which may be attributed to diminished ERG protein. *In vivo*, VCaP xenografts treated with polyamide **1** exhibited reduced growth in a dosage dependent manner, demonstrating its potential as an anticancer therapeutic.

To further examine the mechanism of action for polyamide **1**, we conducted gene expression analysis of VCaP cells after exposure to polyamide **1** in the same time frame as the cytotoxic experiment. Connectivity map analysis of gene expression signatures from treated VCaP cells indicated overlap with expression profiles of several topoisomerase inhibitors. *In vitro* assays for inhibition of both Topo1 and Topo2 confirmed that polyamide **1** is able to attenuate enzymatic activity of both enzymes. Similar results have been reported for other minor groove binders [42–47]. Furthermore, the lack of topoisomerase 2 cleavage complex formation in the inhibition assays suggests polyamide **1** functions by preventing protein-DNA interactions. This mechanism is in contrast to most drugs that target topoisomerases, which poison the enzymes. Drugs such as etoposide, doxorubicin, and camptothecin work by causing covalent adducts, which results in genotoxicity [48].

In addition to inhibition of Topo1 and Topo2, polyamide **1** has been reported to antagonize AR signaling, block RNA polymerase II elongation, and affect DNA replication by impeding helicase processivity [19, 21, 49]. These effects may be related, as inhibition of Topo1 has been shown to lead to RNA polymerase II and DNA polymerase stalling [50], and treatment of prostate cancer cells with topoisomerase inhibitors has been shown to attenuate AR signaling [35, 36, 51, 52]. Taken together, these data suggest that by virtue of targeting DNA and DNA:protein interactions, polyamide **1** may exhibit antiproliferative effects on cancer cells through polypharmacological mechanisms without inducing genotoxic stress.

## Supporting Information

S1 Fig (A) Chemical structure of 1-FITC. (B) Nuclear localization of 1-FITC in VCaP cells are 24 h and 48 h incubation. (PNG)

S2 Fig Mass spectrometry data for Py-Im polyamides used. All polyamides were characterized using high resolution MALDI-TOF. Because this method leads to cleavage of fluorescein, FITC functionalized polyamides were also characterized by liquid chromatography coupled mass spectrometry (LCMS) equipped with a low resolution ionization spectrometer.
(PNG)

S3 Fig Primer sequences for qPCR analysis. Sequences for mRNA analysis without a listed reference (*) were designed using qPrimerDepot (primerdepot.nci.nih.gov), and the single amplification products verified by agarose gel electrophoresis against the 1.1 kN NEB ladder.
(PNG)

S4 Fig Report of two-way ANOVA analysis from Prism software (GraphPad) for Comet assays in VCaP cells. (PNG)

S5 Fig (A) Table of mRNA expression levels for AR-driven genes under DHT-induced conditions in response to 10 μM polyamide 1. VCaP cells were plated at 31k/cm^2^, treated with medium containing 0.1% DMSO (with or without polyamide) and charcoal-treated FBS (CT-FBS) for 72 h followed by induction with 1 nM dihydrotestosterone (DHT) or vehicle for an additional 24 h. *—mRNA expression levels below threshold. (B) mRNA expression data for polyamides at 1 and 10 μM concentration. VCaP cells plated at 31k/cm^2^ were treated with medium containing 0.1% DMSO vehicle (with or without polyamide) for 72h. mRNA levels were measured by qPCR, referenced to *GUSB*, and the polyamide effects compared to vehicle treated samples. Data shown are average of the fold changes (treated/untreated) for three or more biological replicates +/- standard error. (PNG)

S6 Fig Connectivity map analysis of perturbagens that correlate with gene expression changes induced by polyamide 1 in LNCaP cells. Bolded compounds are topoisomerase inhibitors.
(PNG)

S7 Fig Effect of topoisomerase inhibitors on gene expression in VCaP cells. VCaP cells were plated at 31k/cm^2^, incubated for 24 h, and then treated with medium containing 0.1% DMSO (with or without camptothecin or etoposide) and DHT for 16 h. mRNA levels were measured by qPCR, referenced to *GUSB*, and the effects compared to vehicle treated samples. Data shown are the average fold changes (treated/untreated) for three biological replicates +/- standard error.
(PNG)
