# Supplementary figures and images for: Tumor Repression of VCaP Xenografts by a Pyrrole-Imidazole Polyamide
Source: PLoS One. 2015 Nov 16;10(11):e0143161. doi: 10.1371/journal.pone.0143161 (PMC4646452; doi:10.1371/journal.pone.0143161)

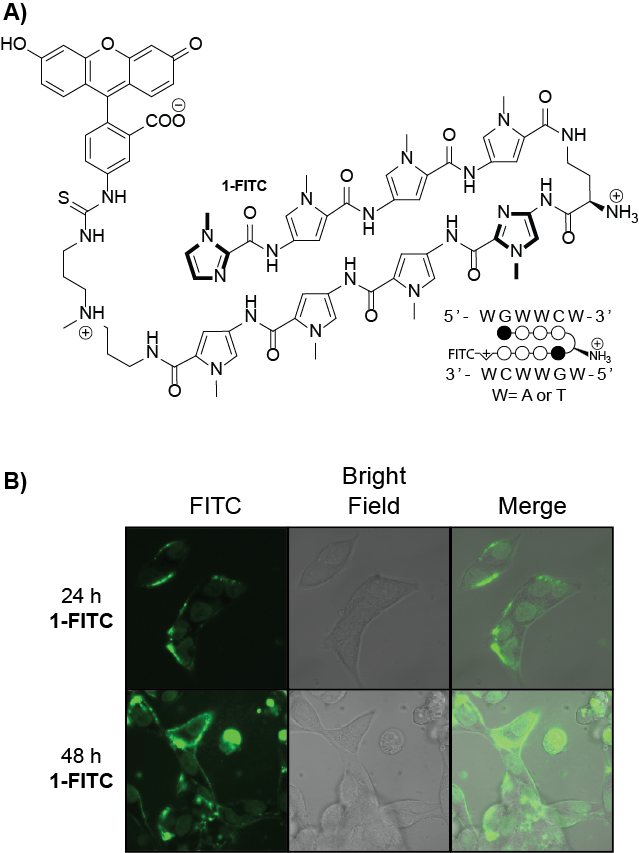

Supplement: S1 Fig — (PNG) [file pone.0143161.s001.png]

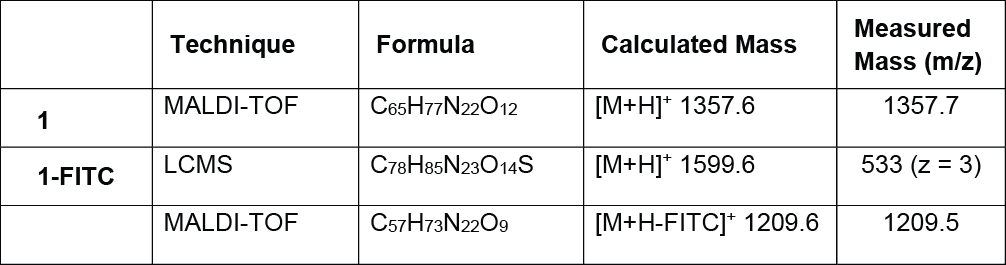

Supplement: S2 Fig — All polyamides were characterized using high resolution MALDI-TOF. Because this method leads to cleavage of fluorescein, FITC functionalized polyamides were also characterized by liquid chromatography coupled mass spectrometry (LCMS) equipped with a low resolution ionization spectrometer. (PNG) [file pone.0143161.s002.png]

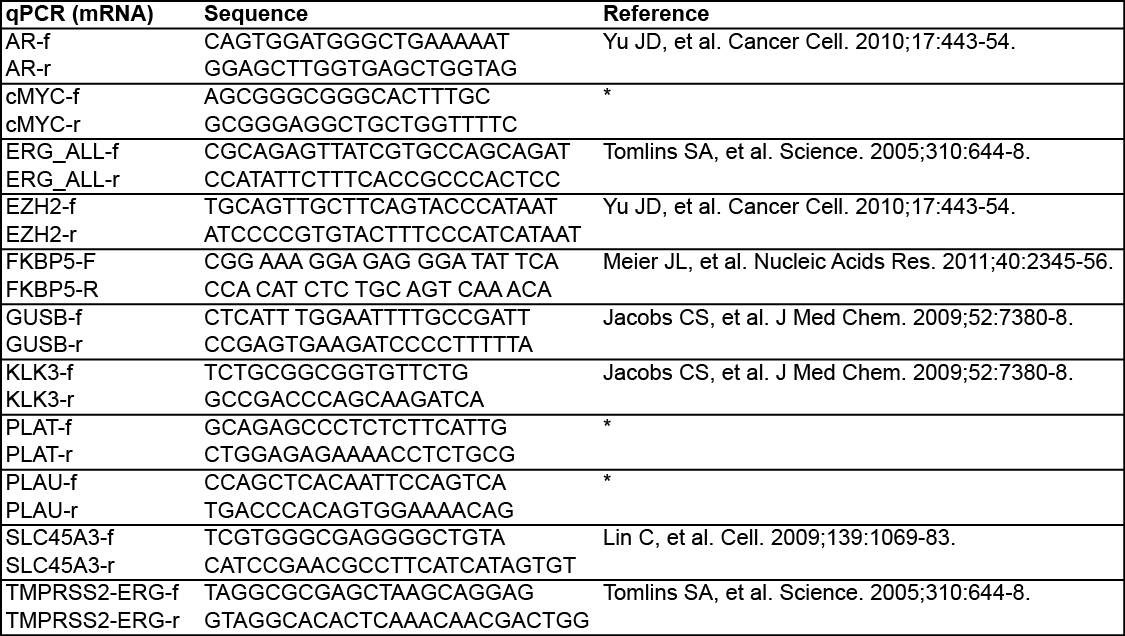

Supplement: S3 Fig — Sequences for mRNA analysis without a listed reference (*) were designed using qPrimerDepot (primerdepot.nci.nih.gov), and the single amplification products verified by agarose gel electrophoresis against the 1.1 kN NEB ladder. (PNG) [file pone.0143161.s003.png]

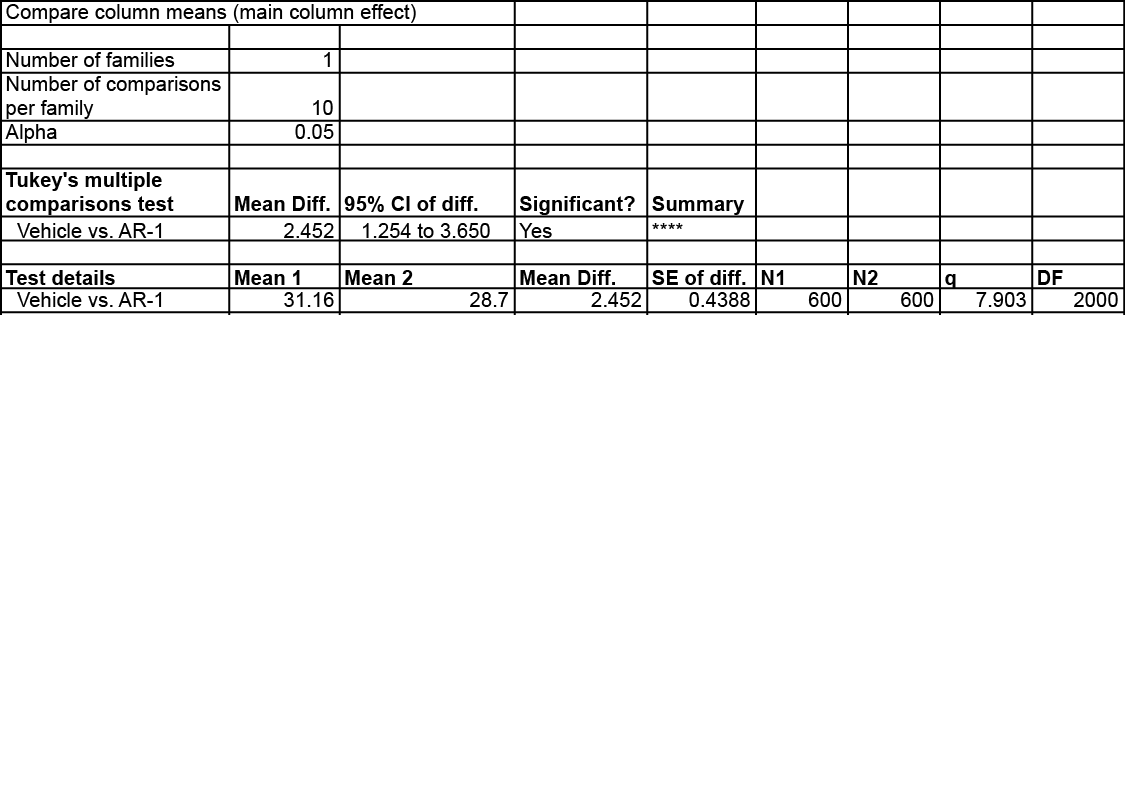

Supplement: S4 Fig — (PNG) [file pone.0143161.s004.png]

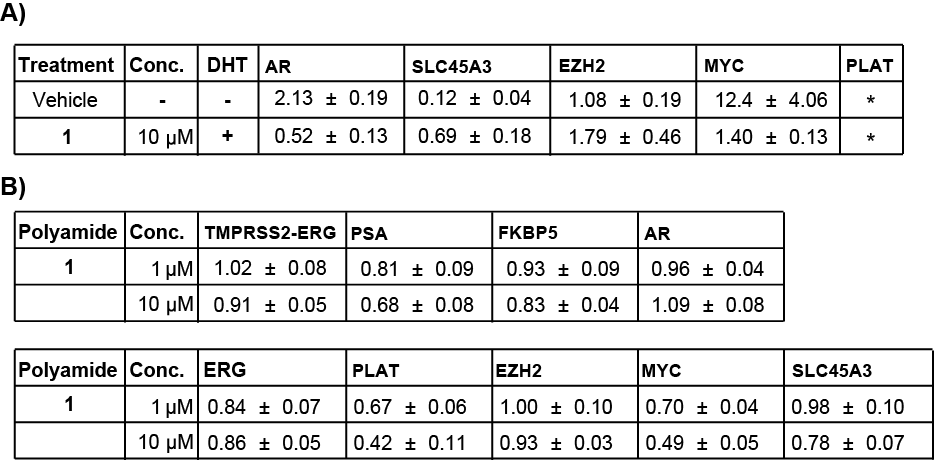

Supplement: S5 Fig — (PNG) [file pone.0143161.s005.png]

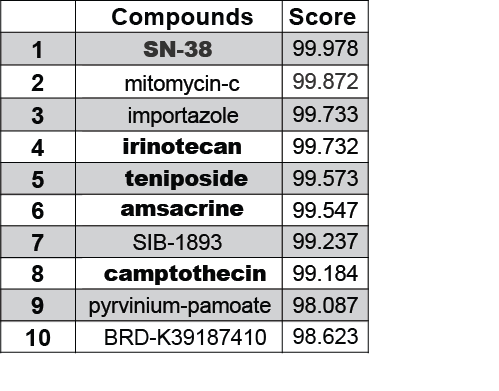

Supplement: S6 Fig — Bolded compounds are topoisomerase inhibitors. (PNG) [file pone.0143161.s006.png]

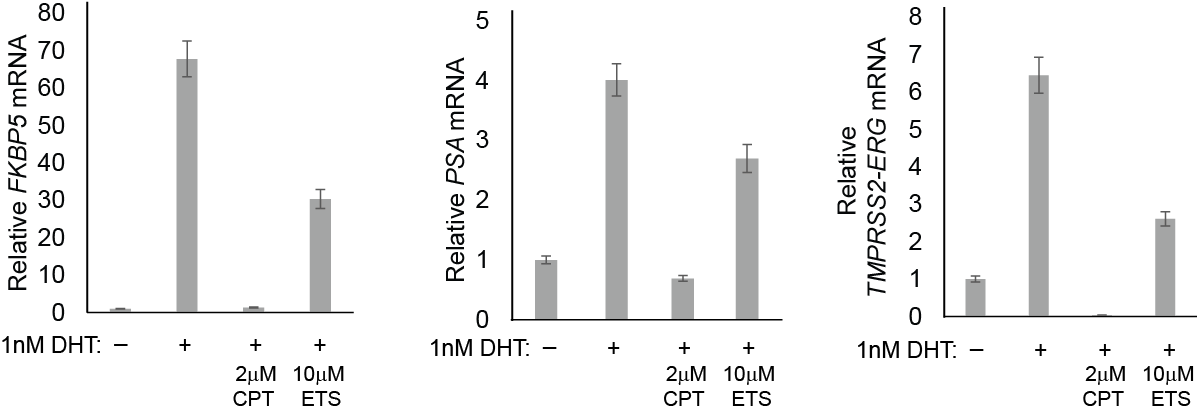

Supplement: S7 Fig — VCaP cells were plated at 31k/cm2, incubated for 24 h, and then treated with medium containing 0.1% DMSO (with or without camptothecin or etoposide) and DHT for 16 h. mRNA levels were measured by qPCR, referenced to GUSB, and the effects compared to vehicle treated samples. Data shown are the average fold changes (treated/untreated) for three biological replicates +/- standard error. (PNG) [file pone.0143161.s007.png]
